# Administration of Recombinant Heat Shock Protein 70 Delays Peripheral Muscle Denervation in the SOD1^G93A^ Mouse Model of Amyotrophic Lateral Sclerosis

**DOI:** 10.1155/2012/170426

**Published:** 2012-08-01

**Authors:** David J. Gifondorwa, Ramon Jimenz-Moreno, Crystal D. Hayes, Hesam Rouhani, Mac B. Robinson, Jane L. Strupe, James Caress, Carol Milligan

**Affiliations:** ^1^Department of Neurobiology and Anatomy, Wake Forest University School of Medicine, Winston-Salem, NC 27157, USA; ^2^The ALS Center, Wake Forest University School of Medicine, Winston-Salem, NC 27157, USA; ^3^Musculoskeletal Research Section, Eli Lilly and Company, Indianapolis, IN 46285, USA; ^4^Torrey Pines Institute for Molecular Studies, Port Street Lucie, FL 34987, USA; ^5^Center for Human Genomics, Wake Forest School of Medicine, Winston-Salem, NC 27157, USA; ^6^Department of Neurology, Wake Forest University School of Medicine, Winston-Salem, NC 27157, USA

## Abstract

A prominent clinical feature of ALS is muscle weakness due to dysfunction, denervation and degeneration of motoneurons (MNs). While MN degeneration is a late stage event in the ALS mouse model, muscle denervation occurs significantly earlier in the disease. Strategies to prevent this early denervation may improve quality of life by maintaining muscle control and slowing disease progression. The precise cause of MN dysfunction and denervation is not known, but several mechanisms have been proposed that involve potentially toxic intra- and extracellular changes. Many cells confront these changes by mounting a stress response that includes increased expression of heat shock protein 70 (Hsp70). MNs do not upregulate Hsp70, and this may result in a potentially increased vulnerability. We previously reported that recombinant human hsp70 (rhHsp70) injections delayed symptom onset and increased lifespan in SOD1^G93A^ mice. The exogenous rhHsp70 was localized to the muscle and not to spinal cord or brain suggesting it modulates peripheral pathophysiology. In the current study, we focused on earlier administration of Hsp70 and its effect on initial muscle denervation. Injections of the protein appeared to arrest denervation with preserved large myelinated peripheral axons, and reduced glial activation.

## 1. Introduction

Amyotrophic lateral sclerosis (ALS) is a neurodegenerative disorder affecting both upper and lower motoneurons (MNs), resulting in gradual muscle weakening and loss of MN function ultimately leading to paralysis and death of afflicted individuals. Much ALS research has focused on MN cell bodies and dendrites within the spinal cord and the role of local glial cells. Many therapeutic interventions have been designed to intervene in pathological events that occur in the anterior horn region with the goal of preventing MN cell body degeneration. However, the death of MNs occurs late in the disease process in mouse models, and even when physical degeneration of the cell is prevented, survival of the animal is only moderately prolonged [1].

More recently, research has focused attention on early events including muscle denervation that begins during the first postnatal month in the SOD1^G93A^ mouse [1–5]. Clinical onset of behavioral pathology is generally considered to occur during the third postnatal month; however, we, and others have observed more subtle behavior changes at earlier time points ([6]; unpublished observations). Furthermore, pathological events such as fragmentation of the Golgi apparatus, vacuolization of mitochondria, deficits in axonal transport, and ER stress are also observed early postnatally [5, 7–10]. Together these results indicate that pathological events in ALS mice occur substantially earlier than previously thought. The etiological event triggering muscle denervation and other pathological events remain undetermined. Although several hallmark pathological features have been identified including increased oxidative stress and reactive oxygen species (ROS) production, glutamate excitotoxicity, protein aggregation, and axonal transport deficits [11], each of these proposed mechanisms can create a stressful environment, compromising the cell's functionality and survival. A common cellular mechanism following stress is the heat shock response, characterized by an increase in the transcription of a subset of genes resulting in the production of inducible heat shock proteins [12–14]. Therefore, examining the heat shock response is a reasonable approach for elucidating the initiation of pathophysiology in ALS.

Previous work in our lab has shown that exogenous delivery of recombinant human heat shock protein 70 (rhHsp70) delays symptom onset and increases lifespan in SOD1^G93A^ mice [15]. Data from this study also revealed that administration of rhHsp70 at postnatal day 30 (P30) extended lifespan longer than beginning administration at P50. Furthermore, the exogenous rhHsp70 was localized to skeletal muscle.

In the present study, the role of exogenous delivery of rhHsp70 on peripheral denervation in the SOD1^G93A^ mouse was investigated in an attempt to further delineate the mechanism of action of rhHsp70. The results revealed attenuated neuromuscular junction (NMJ) denervation in the medial gastrocnemius (MG) and tibialis anterior (TA) muscles. Protein injections also preserved larger myelinated axons in peripheral nerves, and glia cell activation was significantly decreased in treated animals. These data provide additional evidence for the role of rhHsp70 in reducing pathophysiology in SOD1^G93A^ mice.

## 2. Materials and Methods

### 2.1. Animals

All animal experiments were approved by the Wake Forest University Animal Care and Use Committee. Mice from the same colony of a previous paper were used in this study [15]. Wild type females and SOD1^G93A^ males (B6SJL-TgN (SOD1-G93A) 1Gur), obtained from Jackson Laboratory (Bar Harbor, ME), were bred to generate SOD1^G93A^ mice. Mice were weaned and genotyped at P21. DNA was obtained from tail clips and using an alkaline lysis extraction [16]. For this study 18 SOD1 mice treated with rhHsp70, 39 SOD1-nontreated controls, and 36 WT controls were used. For histological analysis of NMJ denervation and glial activation 5 treated, 13 nontreated SOD1, and 10 non-transgenic littermates (wild type (WT)) were used. For determination of axon number and area, 5 treated, 10 nontreated-SOD1, and 10 WT were used. For Western blot analysis 8 treated-16 nontreated-SOD1, and 6 WT were used.

### 2.2. Treatments

Animals were treated with rhHsp70 (Assay Designs, Ann Arbor, MI, catalog #ESP-555; 20 *μ*g diluted in 100 *μ*L sterile saline) similar to a previous study [15]. G93A mice were intraperitoneally injected with rhHsp70 three times weekly beginning at P30. P30 was chosen because it provided the highest lifespan increase in a previous study using the same paradigm [15]. Because mean survival varies between male and female SOD1^G93A^ mice [17], similar numbers of each gender were included in each treatment group. Animals were sacrificed at P30, P75, and P120. WT and untreated SOD1^G93A^ mice served as controls. In our previous study [15] WT mice were treated for nine months with 20 *μ*g rhHsp70 beginning at P50. These animals displayed normal hindlimb splay locomotor ability and weight gain/maintenance. The internal organs showed no gross abnormalities at autopsy. Also in the previous study, intraperitoneal injections of bovine serum albumin (BSA; 20 *μ*g in 100 *μ*L sterile saline) and saline alone were used as negative controls. There were no survival promoting effects of either BSA or saline alone, and there was no statistical difference between the two groups and uninjected controls. For these reasons we only used uninjected controls in the present study.

### 2.3. Hindlimb Neuromuscular Junctions (NMJs)

For counting innervated hindlimb skeletal muscle NMJs, immunohistochemistry was performed on MG and TA muscles. Animals were transcardially perfused with 2% paraformaldehyde in 0.2 M sodium phosphate buffer (PB). The muscles were dissected out, rinsed twice with PBS, and placed in 30% sucrose for at least 72 hours at 4°C. The muscles were sucrose-embedded and cut at 30 *μ*m. Antigen retrieval was achieved using an SDS pretreatment [18] and the sections were stained for the presynaptic vesicular acetylcholine transporter (VAChT; Santa Cruz Biotechnology, Santa Cruz, CA) and postsynaptic localized alpha-bungarotoxin (a-BTX; Invitrogen, Eugene, OR) [19]. Some sections were also colabeled with neurofilament light chain (NF-L; Millipore, Billerica, MA, USA). The percentage of innervated NMJs was determined by counting at least 600 NMJs in each treatment group using previously established counting criteria [15].

### 2.4. Axon Counts and Area

Mice were transcardially perfused with 2% paraformaldehyde and 2% glutaraldehyde in sodium cacodylate buffer. The fourth lumbar ventral root (L4 VR), the sciatic nerve, and the tibial branch of the sciatic nerve were dissected out of each animal. The nerves were refrigerated in the same buffer overnight, postfixed in 2% osmium tetroxide for 1 hour, dehydrated, and embedded in Araldite 502 (Ted Pella Inc, Redding, CA). Myelinated axons were counted in 1 *μ*m toluidine stained sections [1]. Axon area was determined by tracing individual axons at 40X and analyzing them using Scion Imageware (Frederick, MD, USA).

### 2.5. Glial Activation

 Lumbar spinal cords from animals perfused with 2% paraformaldehyde in PBS were analyzed for astrocyte and microglia activation. Spinal cords were dissected, sucrose embedded, and cut at 30 *μ*m. Sections were stained with antibodies against choline acetyltransferase to label MNs (ChAT; Millipore, Billerica, MA) and glial fibrillary acidic protein to label astrocytes (GFAP; Dako, Denmark) or ionized calcium binding adaptor molecule 1 to label microglia (Iba1; Wako Chemical USA, Richmond VA).

To assess glial cell activation, fluorescence pixel intensity was measured using ImageJ software (Frederick, MD). The ventral horn of each section analyzed was centered in a 275 *μ*m × 400 *μ*m rectangle. Representative photomicrographs, encompassing an area of 430 *μ*m × 650 *μ*m, were taken, using a Hamamatsu Digital Camera (model # C4742-95, Bridgewater, NJ) of each analyzed section. The possible gray values and the number of pixels found for each gray value were determined. The mean and modal gray values were calculated for each image. The mean gray scale values were averaged for each animal and group at each time point.

### 2.6. Western Blots

TA, soleus, and MG muscle tissue was dissected from mice at P30 and P75. Protein concentration was determined and 50 *μ*g of muscle was resolved on a 12% polyacrylamide gel. After the protein was transferred to a PVDF membrane (Millipore), the membrane was blocked overnight at 4°C in 5% milk prepared in TBS-T. The following day the membrane was incubated with primary antibody overnight, at 4°C. The primary antibody used was anti-Hsp70 (SPA-820 Assay Designs). Mouse anti-actin (MAB1501, Calbiochem) was used to verify equal loading.

Endoplasmic reticulum (ER) stress has been identified as an early marker of MN stress in our mouse model [5]. An indicator of ER stress is an increase in immunoglobulin heavy chain-binding protein (BiP) levels [20]. ER stress was assayed using a rabbit anti-BiP antibody (C50B12, Cell Signaling). Western blotting was performed as previously described. Mouse anti-actin (MAB1501, Calbiochem) was also used to verify equal loading.

The secondary antibody, HRP-conjugated donkey anti-mouse IgG or donkey anti-rabbit IgG (Jackson Immuno), was applied for 1.5 hours at room temperature. The membrane was washed in TBS-T and developed using the Super Signal West Pico ECL kit (Pierce). Densitometry analysis was performed to quantify the results.

### 2.7. Data and Statistical Analysis

All samples for determination of NMJ denervation, glial activation, or axon number/area were coded prior to analysis and the individual performing the analysis was blinded to the code. Statistical analysis (Graph Pad Prism 5) was performed by generating a mean value followed by formulating the standard error of the mean. Statistical significance of spinal cord fluorescence was determined using a two way ANOVA at P30 and a repeated-measures (mixed model) ANOVA at P75 and P120 on all of the measured values above baseline. Statistical significance of myelinated axons and NMJs was determined by running an ANOVA, followed by the Tukey-Kramer multiple comparisons post-hoc test.

## 3. Results

### 3.1. rhHsp70 Slows NMJ Denervation

Denervation of neuromuscular junctions begins during the fourth postnatal week in the SOD1^G93A^ mouse model of ALS [1]. Preventing or delaying early pathological events such as muscle denervation may extend survival as suggested by previous studies where treatments beginning at day 30 resulted in the largest increase in lifespan [15, 21]. Accordingly, in this study, treatment with rhHsp70 was started at P30.

**Figure 1:**
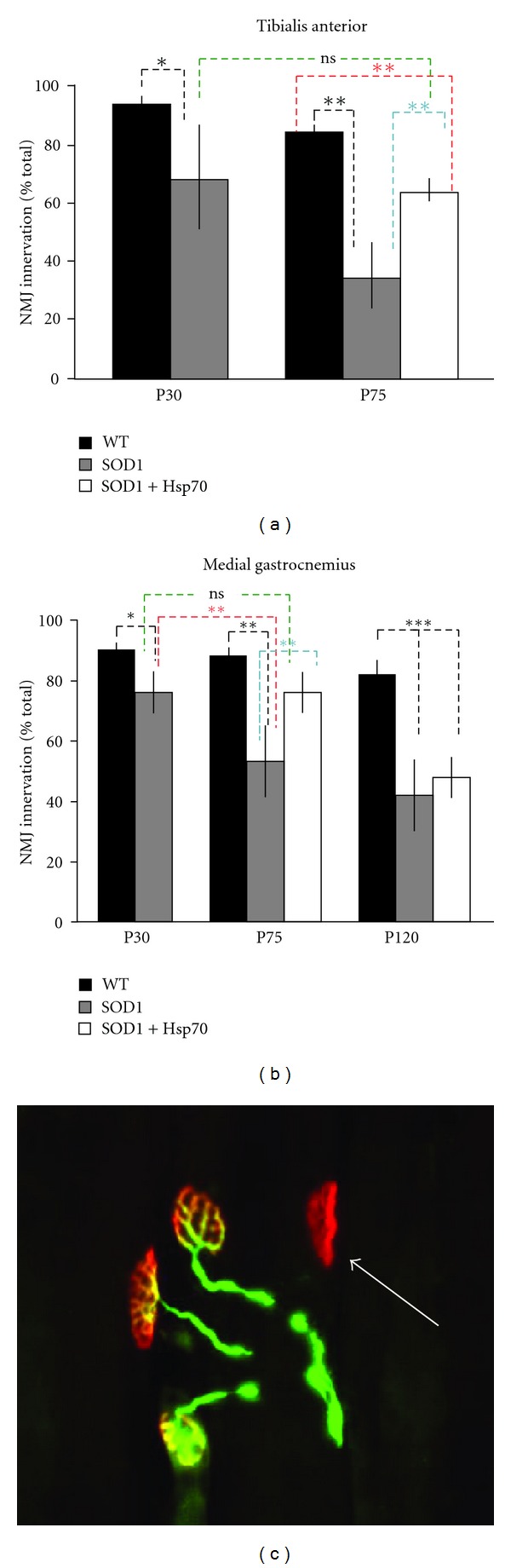
rhHsp70 injections maintain NMJ innervation. (a) Denervation of TA NMJs in SOD1 is evident by P30 and is further increased by P75. Treatment with rhHsp70 appeared to attenuate this denervation. Data are represented as percent of WT ± SEM at each time point. Statistical significance was determined by running an ANOVA, followed by the Tukey-Kramer multiple comparisons posthoc test (*P < 0.05; **P ≤ 0.01 and ***P ≤ 0.001). (b) The MG in SOD1 mice displayed significant denervation at P30 and there is further denervation at P75 as compared to WT mice. rhHsp70 treatment maintained innervation of MG NMJs between P30 and P75. Statistical analysis was performed as described in (a) P30 WT TA *n* = 9; P30 SOD1 TA *n* = 8; all other groups *n* = 5. (c) Photomicrograph to illustrate criteria for innervated and denervated NMJs at P75. Colocalization of *α*BTX-labelled nAChR clusters (red) with VAChT and neurofilament-L nerve terminals (green) indicate innervated NMJs. The arrow denotes a denervated NMJ because of the lack of VAChT labeling.

By P30 significant denervation has already occurred in the tibialis anterior (TA) and medial gastrocnemius (MG) muscles (Figure 1). The TA muscle contains Type 2b fibers (FF) and type 2a (FR) fibers, with the anterior portion of the muscle expressing almost exclusively type 2b fibers, whereas MG contains a mixture of fast (type 2a and 2b) and slow (type 1) fibers [22, 23]. Motoneurons that innervate type 2b fibers appear to be highly susceptible in ALS mice [22, 23]. In untreated SOD1 animals, the extent of denervation increases between P30 and P75. In rhHsp70 treated mice there was a higher percentage of innervated NMJs as compared to untreated animals. Surprisingly, there was no difference between the extent of NMJ innervation in rhHsp70-treated P75 SOD1 mice as compared to muscles from P30 SOD1 mice (Figures 1(a) and 1(b)). While we had previously shown that administration of rhHsp70 can reduce NMJ denervation upto P90 in the MG [15], by end stage (P120), there was no difference in NMJ innervation between treated and untreated animal (data not shown). From these data, it appears that treatment delays but does not prevent muscle denervation, possibly because the mechanisms by which rhHsp70 is protective are overtaken by other debilitating events.

### 3.2. rhHsp70 Maintains Peripheral Nerve Axon Morphology

**Figure 2:**
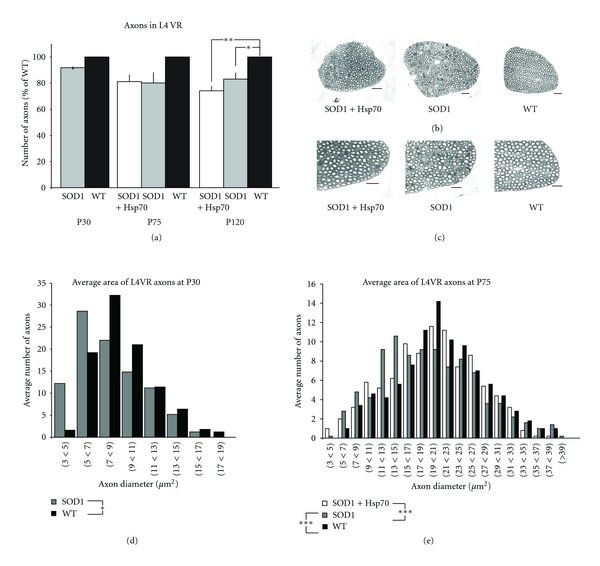
rhHsp70 injections preserve larger axons in the L4VR. (a) There was no difference in the overall number of myelinated axons in the 4th lumbar ventral root at P30 or P75 although there appears to be a trend toward decreased numbers in the SOD1 groups at P75. Data are represented as percent of WT ± SEM at each time point. *N* = 5 for each group. Statistical significance was determined by running an ANOVA, followed by the Tukey-Kramer multiple comparisons post-hoc test. (b) Although there was not a significant difference in the number of axons in treated and untreated mice, photomicrographs suggest that disease morphology of myelinated axons has progressed more in untreated mice as compared to treated animals. Photomicrographs of myelinated axons were taken at 40x. Scale bars = 40 *μ*m. (c) Enlargements of photomicrographs of myelinated axons taken at 40x. Scale bars = 40 *μ*m. (d) Size profiles of the axon area revealed a greater number of larger axons in WT animals (*P < 0.05). Data are representative histograms of the average area of myelinated axons in the L4VR of SOD1-untreated and WT mice at P30. Statistical significance was determined by running a two-way ANOVA. Axons were traced using a camera lucida at 40x scanned and average axon area was determined using Scion Image software. Data are represented as the average number of axons per area range. *N* = 5 for each group. (e) There were a greater number of larger axons in SOD1-treated and WT animals as compared to SOD1-untreated mice (***P < 0.001) at P75. There was no significant difference in the number of larger axons between SOD1-treated and WT animals. Data are representative histograms of the average number of axons per area range. *N* = 5 for each group at P75. Statistical significance was determined using repeated measures (mixed model) ANOVA. Statistical significance was determined using repeated measures (mixed model) ANOVA.

Recent evidence derived from the study of animal models of various neuropathological conditions has revealed that damage to axons and synapses often long precedes the activation of death pathways and the onset of clinical (i.e., functional) pathology [24–27]. Since MNs innervate skeletal muscles through their long axons that travel through peripheral nerves, changes in the number and/or morphology of axons in the nerves might be expected. Therefore, three regions of nerves that innervate hindlimb muscles, L4 ventral root (VR), and sciatic and tibial nerve branches were examined. We did not detect a significant difference in the number of myelinated axons between WT, SOD1-treated, or SOD1-untreated mice L4 VR at P30 or P75 (Figure 2(a)). However, while absolute number of axons was not affected, there were clear differences in the morphology of the VR at P75 (Figure 2(b)). The VR from rhHsp70-treated animals appeared similar to that from WT mice, while the VR of untreated SOD1 mice showed exhibited smaller myelinated axons, degenerating profiles, and cellular infiltration. At P120 there was a significant difference between the number of axons in WT and untreated SOD1 mouse groups, but no difference between treated and untreated SOD1 animals (data not shown). In contrast to number of axons, as early as P30, the size of large myelinated fibers was reduced in untreated SOD1 mice as compared to WT (Figure 2(c)). At P75 there was no difference in the size of VR axons between WT and rhHsp70-treated animals, but there was a significant decrease in the size of VR axons in untreated SOD1 animals. The peak number of axons in WT and treated SOD1 animals had a diameter of 19–21 *μ*m and untreated SOD1 animals had a peak number of axons that had a diameter of 13–15 *μ*m (Figure 2(d)). P120 WT mice had a greater number of large axons than both SOD1 groups at P120, but there was no difference between the two SOD1 groups at this age (data not shown).

**Figure 3:**
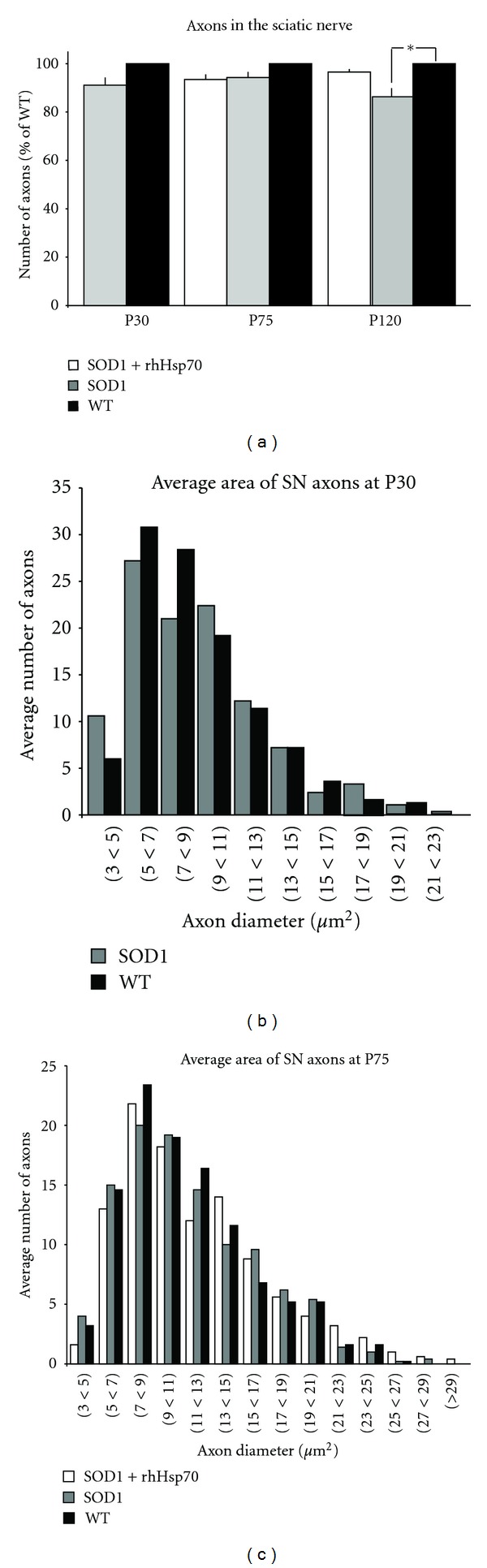
There is no change in number or size of axons in sciatic nerve with rhHsp70 treatment. (a) There was no difference in the overall number of myelinated axons in the sciatic nerve at P30 or P75. Data are represented as percent of WT ± SEM at each time point; *n* = 5 for each group. Statistical significance was determined as previously described. (b) Size profiles of the axon area done at P30 revealed there was no difference in the number of larger axons in SOD1 mice. Axon area and statistical significance were determined as previously described. (c) Data are representative histograms of the average area of myelinated axons in the sciatic nerve of each group at P75. There was not a significant difference in average area number of axons between the groups at this age. Axon area and statistical significance were determined as previously described.

In the mouse, the motor component of the sciatic nerve originates predominantly from L3-4 [28] and innervates posterior thigh and leg muscles. The number and average area of myelinated axons were examined anterior to the point where the sciatic nerve divides into the peroneal and tibial branches. There was no difference in the absolute number or size of axons between WT, treated or untreated SOD1 animals at any age examined (Figure 3 and data not shown).

**Figure 4:**
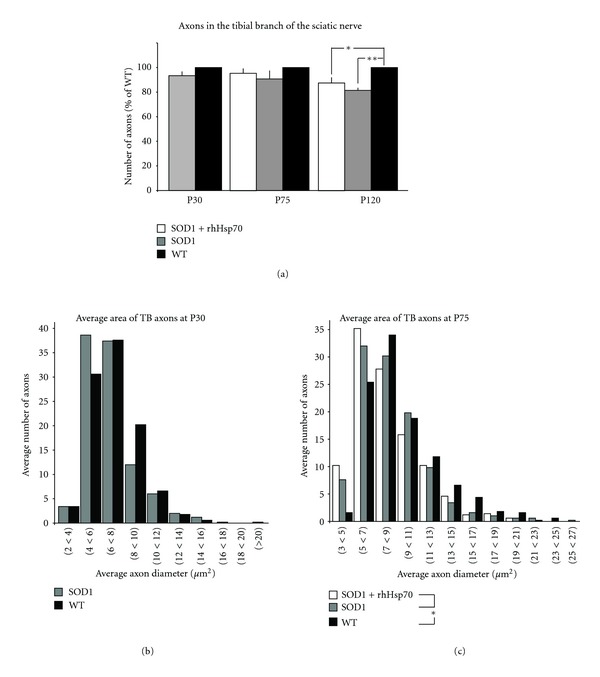
rhHsp70 slows axon atrophy in the tibial branch of the sciatic nerve. (a) There was no difference in the overall number of myelinated axons in the tibial branch of the sciatic nerve at P30 or P75. Data are represented as percent of WT ± SEM at each time point; *n* = 5 each group. Statistical significance was determined as previously described. (b) Size profiles of the axon area at P30 revealed that there are comparable numbers of larger myelinated axons between WT and SOD1-untreated animals. Axon area and statistical significance were determined as previously described. (c) Axon area analysis determined that only WT animals had a greater number of larger myelinated axons in the tibial branch of the sciatic nerve at P75 (*P ≤ 0.05). Axon area and statistical significance were determined as previously described. Axon area was determined as previously described.

 Axons in the tibial branch of the sciatic nerve were also analyzed in a similar fashion as in the L4 VR and sciatic nerve. This branch of the sciatic nerve provides innervation to the MG. As with the other segments, there was no significant difference in the number of myelinated axons between groups at any time examined (Figure 4(a)), nor was there difference in diameter size between WT and SOD1 at P30 (Figure 4(b)). Axon area analysis revealed a higher number of large myelinated axons in the WT mice as compared to SOD1 groups at P75 or P120 (Figure 4(c) and data not shown). Although the difference in axon diameter at P75 between SOD1 and WT reached statistical significance, the peak number of axons in WT had a diameter of 7–9 *μ*m whereas the SOD1 groups had a peak number of axons with a diameter of 5–7 *μ*m. These data suggest that there is a little change in the number or size of axons in more distal nerves between SOD1 and WT animals. Furthermore, treatment with rhHsp70 appears to maintain axon health in the VR where differences between SOD1 and WT are detected.

### 3.3. rhHsp70 Reduces Glial Activation

**Figure 5:**
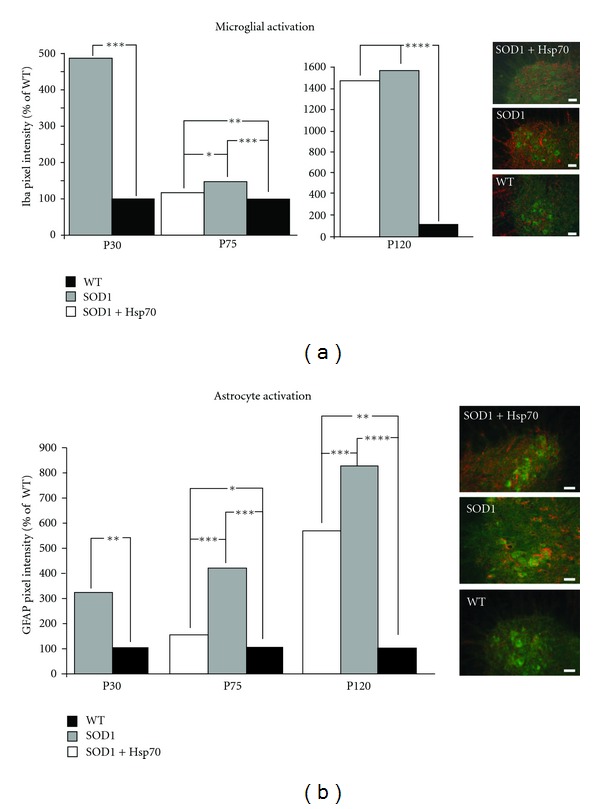
rhHsp70 injections attenuate early glial cell activation. (a) The fluorescent pixel intensity of Iba1 staining for microglia was increased in the SOD1 versus WT animals at P30 and P75. Treatment with rhHsp70 appeared to reduce the expression of Iba1 at P75, but the levels were greater than WT animals. Pixel intensity was measured using Image J software. Each fluorescent pixel in the section was measured and the values were compiled and averaged across each treatment group. Histograms represent the average number of pixels in a given range of arbitrary intensity units presented as % WT. Statistical significance was determined using a two-way ANOVA at P30 and a repeated measure (mixed model) ANOVA at P75 on all of the measured values above baseline (***P ≤ 0.001; **P ≤ 0.01; *P ≤ 0.05). *N* = 4 for each group. Images of the lateral motor column of P75 mice that were treated with Hsp70, untreated, or WT are shown on the right. 30 *μ*m spinal cord sections were stained with Iba-1 (red) and ChAT (green). Scale bars = 20 *μ*m. (b) GFAP expression was the greatest in SOD1 animals as compared to WT at P30 and P75. Treatment with rhHsp70 substantially reduced the expression of GFAP in SOD1 animals. Quantification of fluorescence and statistical analysis was performed as described above. Images of the ventral horn of P75 mice that were treated with Hsp70, untreated, or WT are shown on the right. 30 *μ*m spinal cord sections were stained with GFAP (red) and ChAT (green). Scale bars = 20 *μ*m.

ALS MN pathology is accompanied by astrocyte and microglia activation [5, 28–31]. Increased microglia activation is an event that has been shown to occur both early and near end stage in SOD1^G93A^ mice [5, 28]. At P30, there was a five-fold increase in microglial activation as detected by Iba1 immunofluorescence levels in SOD1 versus WT lumbar spinal cords (Figure 5(a)). Interestingly, there was an apparent decrease in microglial activation in our untreated P75 SOD1 mice as compared to P30 animals. Treatment with rhHsp70 beginning at P30 resulted in reduced microglial activation at P75, although the level of activation at this stage is much lower as compared to earlier and later time points. Microglial activation was observed in close proximity to MNs as opposed to throughout the spinal cord, in SOD1 animals (Figure 5(b)). At P120 both treated and untreated SOD1 groups exhibited microglial activation at comparable levels to that observed at P30 (data not shown).

 Dramatic changes in astrocyte activation have been reported between P80 and P100 [3, 28], and as early as P40 in another report in ALS mice [1]. In the current study astrocyte activation was detected by increased GFAP immunohistochemistry as early as P30 in SOD1 versus WT spinal cord. At P75 a similar level of activation as seen at P30 was observed in SOD1-untreated animals; however, activation was reduced in treated animals versus untreated SOD1 mice (Figure 5(a)). As with microglia, activated astrocytes were observed in the region of the ventral horn. (Figure 5(b)). By P120 the extent of astrocyte activation was similar in both SOD1 groups of mice (data not shown).

### 3.4. rhHsp70 Has No Effect on Bip Levels in the Lumbar Spinal Cord

**Figure 6:**
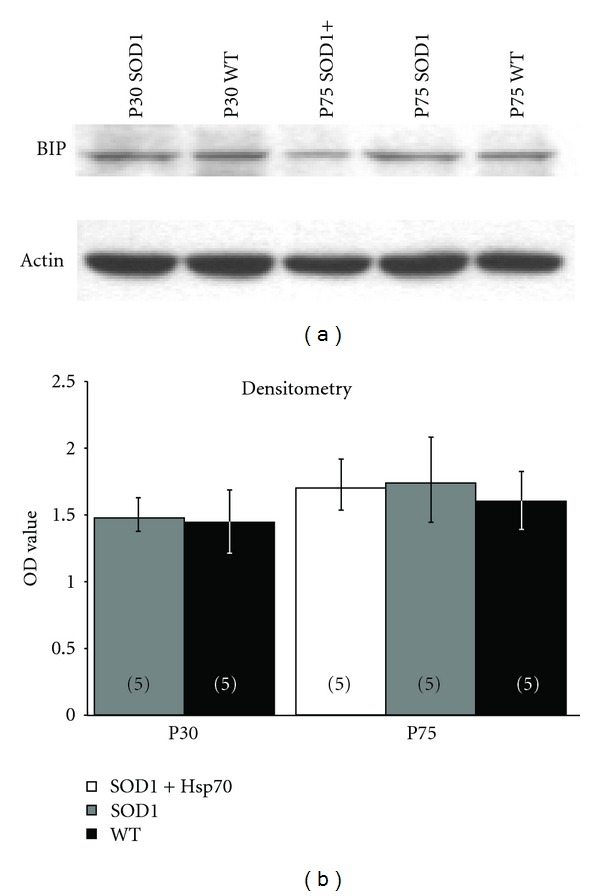
Treatment with rhHsp70 has no effect on ER stress in the spinal cord as determined by BiP expression. (a) Protein extracts were collected from individual lumbar spinal cords and subjected to electrophoresis on a 12% SDS PAGE gel. Proteins were transferred to Immobilon-P membrane, blocked in TBS-T, incubated with a BiP antibody (Cell Signaling) and a donkey anti-rabbit secondary antibody (Jackson Immuno), reacted in ECL, and exposed to X-ray film. (b) Relative levels of BiP were determined using densitometry (*n* = 5 blots for each spinal cord). There was only a slight increasing trend in relative levels of BiP when comparing SOD1-untreated to SOD1-treated and WT mice. Statistical significance was determined using a two-way ANOVA followed by Tukey-Kramer post hoc test.

 Gene expression profiling revealed that vulnerable MNs in the SOD1 mouse exhibit increased components of the ER stress response [5]. Increased expression of BiP, an ER luminal protein, is a hallmark indicator of ER stress and was shown to have increased expression in vulnerable MNs as early as day 28 in SOD1^G93A^ mice. To determine if administration of rhHSP70 affected BiP expression, lumbar spinal cord protein extracts were examined by Western blot at P30 and P75 (Figure 6). There was no detectable difference between BiP levels between WT and SOD1 mice at P30, nor was there a significant difference in BiP levels between groups; treatment with Hsp70 did not affect expression of BiP as compared to untreated group.

### 3.5. Hsp70 Expression Varies in Different Hindlimb Skeletal Muscles

**Figure 7:**
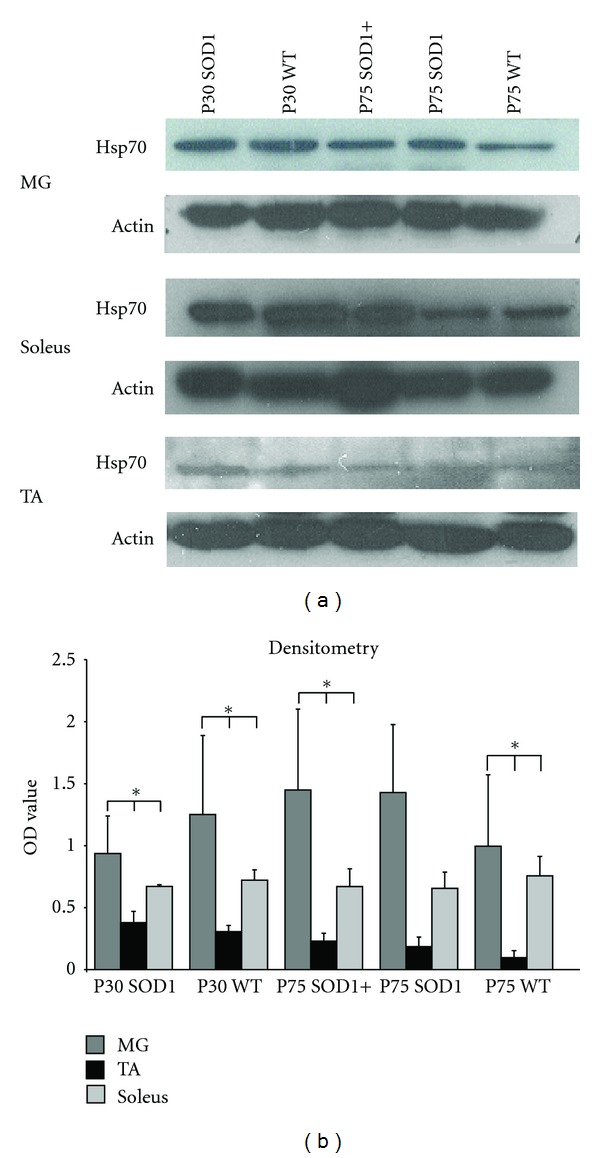
Muscles with type 1 fibers have increased expression of Hsp70 as compared to muscles with predominantly Type 2 fibers. (a) Images of representative Western blots probed for Hsp70 or actin (loading control). (b) Quantification of Hsp70 expression was performed with densitometry analysis confirmed increased expression of Hsp70 in muscles containing type 1 fibers. There was no significant difference in Hsp70 expression between WT and SOD1 muscles and treatment with rhHsp70 did not affect this expression. Statistical significance was determined using a two-way ANOVA followed by Tukey-Kramer post hoc test (*n* = 3  *P < 0.05).

 Muscle cells can secrete Hsp70 and Hsp70 appears to be critical for muscle extract's survival promoting activity for MN in culture [29]. TA muscle contains type 2a and 2b fibers, and the MG contains a mixture of fast and slow fibers [22, 23]. These muscles undergo early NMJ denervation ([1, 23]; Figure 1). The soleus muscle contains predominantly slow-type fibers and undergoes little denervation even at late-stage ([22, 23]; Milligan and Oppenheim labs, submitted). The different fiber types may exert different survival promoting activity, and for this reason the expression of heat shock proteins was examined. The overall level of Hsp70 was significantly greater in the soleus and MG muscles, and lowest in the TA that expresses only fast-type fibers (Figure 7). While there was no change in expression of Hsp90, expression of Hsp40 showed an opposite expression pattern compared to Hsp70 (not shown). Treatment with rhHsp70 did not appear to alter the levels of Hsps expression in any of the muscles examined (Figure 7 and not shown).

## 4. Discussion

A prominent clinical feature of ALS is muscle weakness due to lower MN dysfunction and degeneration resulting in denervation of the NMJ. Muscle strength correlates well with function and survival in ALS [32] and ALS patients with predominantly upper MN symptoms live significantly longer than those with more lower MN (LMN) dysfunction [33]. Therefore, preserving functional integrity of the entire lower neuromuscular system (MNs, axons, and NMJs) may lead to improved function and survival even in the face of continued upper MN (UMN) degeneration. In patients with ALS, clinical weakness (weakness that affects function and can be demonstrated on routine neurological examination) does not develop until 30–50% of the MNs innervating that muscle have degenerated [34]. In ALS mouse models muscle denervation and NMJ abnormalities occur months before overt clinical disease onset or MN death [2–4, 23, 35–39]. Indeed, our study confirms a previous report that significant NMJ denervation occurs as early as the first postnatal month [1]. This early denervation correlates with behavioral deficits such as altered paw placement and grip strength ([23], and Milligan, Oppenheim submitted), righting behavior and movement patterns [6, 40, 41]. While we did not perform behavioral analysis in this study, we found that administration of rhHsp70 substantially delays early muscle denervation such that the level of muscle innervation present at the start of treatment (P30) was maintained for 1.5 months in treated animals whereas it significantly decreased in untreated mice over this same period. Preventing or delaying these as well as other early pathological events may be more relevant in terms of therapeutic efficacy, whereas prevention of late events, such as MN death has been shown to be relatively ineffective [1]. Our study provides further evidence that NMJ maintenance is necessary for survival and might be a viable therapeutic target.

We also determined whether rhHsp70 treatment provided protection to peripheral axons together with the maintenance of muscle innervation. Although there were no differences in the number of axons between SOD1 (treated or untreated) and WT animals at P30 or P75, there was an increase in the size of myelinated fibers in Hsp70-treated as compared to untreated SOD1 animals in the ventral roots where the peak axon diameter in treated animals was approximately 6 *μ*m larger than untreated animals. The morphology of axons in L4VRs at P75 of treated mice more closely resembled those in WT mice, compared to obvious signs of degeneration in untreated SOD1 mice. The maintenance of VR axonal integrity is in agreement with our previous observations that administration of rhHsp70 maintained MN soma size and MN number for a longer period of time as compared to untreated animals [15]. At P90 there is an increased number of healthy MNs in rhHsp70-treated animals as compared to untreated SOD1^G93A^, and at P120, while there was no difference in the absolute number of MNs, the soma size was significantly larger in treated animals [15].

Glial activation has been shown to occur in step with ALS progression. Numerous studies have provided evidence of increased glial activation prior to MN death in ALS [28, 30, 42–48]. Activation can be characterized by an increase in the number and shape of microglia and astrocytes in a given area. Along with their increase in numbers and altered morphology, glia also secretes both beneficial and toxic factors that act on MNs [30, 44, 46, 49]. Using immunohistochemistry we find that there is an apparent activation of microglia and astrocytes as early as P30 in the ventral horn of SOD1 mice, whereas glia in other regions of the spinal cord appear to be unaffected. Microglial activation appeared to subside by P75 and reappear at end stage, whereas astrocyte activation in SOD1 mice appeared to increase with disease progression. These results indicate distinct responses by microglia and astrocytes to MN pathology. Treatment with rhHsp70 reduced activation of glia; however, by end stage there was no difference between treated and untreated animals.

This evidence suggests that rhHsp70 treatment alters glial activation which is important in maintaining MN axon function [50]. Delaying glial activation using minocycline has been shown to increase lifespan in SOD1 mice [51–54]. Interestingly, the administration of minocycline to late-stage symptomatic mice exacerbated gliosis [55]. Taken together, these data suggest that the timing of glial activation may be critical for mediating a protective versus adverse effect, and that early suppression of glial activation is necessary to achieve increased lifespan. In our studies, there was no evidence of exogenous rhHsp70 entering the CNS rather it was localized to skeletal muscle [15]. The apparent suppression of glial activation may be an indirect result of rhHsp70's protective effect at the NMJ.

Hsp70 interacts with the DnaJ-like chaperone cysteine string protein (Csp) at the NMJ to ensure the proper release of neurotransmitter [56, 57]. In our previous study, we examined whether rhHsp70 interacts with Csp as a mechanism to promote NMJ innervation, but the data did not support this idea [15], and the present results are supportive of this (not shown). ER stress has been shown to be elevated in SOD1 MNs and may be one of the underlying causes of MN stress and eventual death [5, 58, 59]. Results from the current study suggest a slight increasing trend of Bip/GRP78 levels in spinal cord homogenates, but expression levels of BiP/Grp78 were not significantly different in WT versus SOD1 mice and administration of rhHsp70 did not appear to alter Bip/GRP78 expression. We should note, however, that our results are not necessarily inconsistent with previous studies, because we examined BiP levels in spinal cord lysate whereas the previous studies utilized single cell analysis of MNs (e.g., [6]).

Hsp70 levels have been shown to be greater in type I muscle fibers compared to type II muscles [60, 61]. Quantification of Western blot analysis suggests the TA has the lowest Hsp70 levels of the muscles examined. Additionally, the TA also showed decreased expression of Hsp90 and increased expression of Hsp40. These results are intriguing because administration of Hsp70 to motoneurons in culture is protective, while administration of recombinant Hsp40 resulted in the death of the cells [29]. Because MNs that innervate type 2B fibers are most susceptible in ALS, the apparent reduced expression of Hsp70 in the TA, a muscle that has predominantly type 2B fibers, may limit availability of Hsp70 at the NMJ that may contribute to the susceptibility of the MNs innervating those muscle fibers. Muscle cells have been shown to be able to secrete Hsp70 and Hsp70 appears to be a critical component of muscle extract's MN survival promoting activity [29].

The mechanism by which administration of rhHsp70 maintains NMJ innervation and MN survival in our ALS mice is not known. Unfortunately, at this time, this line of investigation is constrained by the prohibitive cost of the recombinant Hsp70 protein; however, taken together with other studies, our results provide some guidance for future mechanistic studies. Although Hsp70 overexpression has been shown to be beneficial in rodent Alzheimer's disease models [62], when Hsp70 was ubiquitously over-expressed in SOD1 mice, there was no protective effect [63]. These data appear difficult to reconcile with our results; however, one must consider the potential different mechanisms of action of intracellular versus exogenous protein. By crossing SOD1 with mice that chronically expressed inducible Hsp70 at ten times the level of endogenous protein resulted in increased intracellular concentrations in all cells both in CNS and peripheral tissues [63]. Increased intracellular expression of Hsp70 may not be sufficient to promote cell survival. We were able to localize the recombinant protein in muscle (and other peripheral tissues such as liver) and not in the spinal cord or brain. Furthermore, when rhHsp70 is injected into muscle of developing chick embryos, it was not observed internalized in axons or MN soma [29]. While the amounts of rhHsp70 in MNs may be below the level of detection, an alternative hypothesis is that the administered protein is acting via an extracellular mechanism at the NMJ. Biochemical changes within muscle or activation of complement at the NMJ have been suggested to contribute to NMJ denervation [64, 65]. Extracellular rhHsp70 may bind to potentially toxic molecules such as complement to prevent detrimental effects. Alternatively, it may bind to trophic factors to promote their survival promoting activity at the NMJ. These possibilities are the subject of future investigations and may provide novel insight into the mechanism that underlie initial denervation in ALS. Administration of rhHsp70 was not effective at late or end stage of the disease (P120). One possible explanation may be that other disease processes or cell nonautonomous effects not responsive to rhHsp70 have a larger role at this stage. Alternatively, different doses of rhHsp70 may be required at different stages of the disease.

## 5. Conclusions

The major findings of the present study provide evidence that exogenous rhHsp70 affects both central and peripheral disease processes in SOD1^G93A^ mice. The data suggest that larger myelinated axons in L4 VR and NMJ innervation in the TA and MG are maintained until P75 with rhHsp70 injections. Also at the same time point, glia activation, assayed by measuring the amount of fluorescence above of background, is reduced.

Currently, the mechanism of action for rhHsp70 is unidentified. Our findings and the results of previous studies have resulted in two prevailing hypotheses, the administered protein is acting via an extracellular mechanism at the NMJ or it may bind to trophic factors to promote their survival promoting activity at the NMJ. Future studies aimed at the NMJ will provide novel insight into how and if the protein is acting locally at the NMJ.
